# Modification of NFA-Conjugated Bridges with Symmetric Structures for High-Efficiency Non-Fullerene PSCs

**DOI:** 10.3390/polym11060958

**Published:** 2019-06-02

**Authors:** Qiuchen Lu, Ming Qiu, Meiyu Zhao, Zhuo Li, Yuanzuo Li

**Affiliations:** 1 College of Science, Northeast Forestry University, Harbin 150040, China; qiuchenlu1997@126.com (Q.L.); qm15765526850@163.com (M.Q.); 17733720476@163.com (Z.L.); 2 School of Chemistry and Chemical Engineering, Harbin Institute of Technology, Harbin 150001, China

**Keywords:** non-fullerene polymer solar cells, non-fullerene acceptor, small molecules, DFT, D/A interface, voltage loss, fill factor

## Abstract

As electron acceptors, non-fullerene molecules can overcome the shortcomings of fullerenes and their derivatives (such as high cost, poor co-solubility, and weak light absorption). The photoelectric properties of two potential non-fullerene polymer solar cells (PSCs) PBDB-T:IF-TN (PB:IF) and PBDB-T:IDT-TN (PB:IDT) are studied by density functional theory (DFT) and time-dependent DFT (TD-DFT). Based on the optimized structure of the ground state, the effects of the electron donor (D) and electron acceptor (A) (D/A) interfaces PBDB-T/IF-TN (PB/IF) and PBDB-T/IDT-TN (PB/IDT) are studied by a quantum-chemical method (QM) and Marcus theory. Firstly, for two non-fullerene acceptors (NFAs) IF-TN and IDT-TN, the NFA IDT-TN has better optical absorption ability and better electron transport ability than IF-TN. Secondly, for the D/A interfaces PB/IF and PB/IDT, they both have high optical absorption and electron transfer abilities, and PB/IDT has better optical absorption and lower exciton binding energy. Finally, some important parameters (open-circuit voltage, voltage loss, fill factor, and power conversion efficiency) are calculated and simulated by establishing the theoretical model. From the above analysis, the results show that the non-fullerene PSC PB:IDT has better photoelectric characteristics than PB:IF.

## 1. Introduction

With the depletion of traditional fossil energy and serious environmental pollution, it is urgent to find new, clean, and sustainable energy. Solar energy is an excellent clean, sustainable, and new energy, and solar cells are important devices for the efficient collection and utilization of solar energy [1,2,3,4,5,6]. Among them, polymer solar cells (PSCs) have attracted wide attention because of their excellent properties, such as their small weights, low risk of pollution, high photoelectric conversion efficiencies (PCE), and the fact that they are easily manufactured on a large scale [7,8,9,10]. The energy conversion process of PSCs is divided into four basic steps [11,12]: (1) Sunlight excites the active layer molecules, and excitons are formed in the active layer; (2) the excitons are diffused to the interfaces of the electron donor (D) and the electron acceptor (A) (D/A interfaces); (3) the excitons undergo charge separation at the D/A interfaces, generating electrons and hole carriers; (4) charges are transferred to the electrodes to generate current. In order to convert light into electricity more effectively, the active layer molecules of PSCs should have characteristics well-matching with the sunlight spectrum, efficient charge transfer, the ability to produce many carriers, and lower loss of the carriers. The active layer, as an important part of the PSCs, is generally composed of electron donors and electron acceptors [13], which are intermingled or formed into a bilayer structure. Fullerenes and their derivatives are the typical electron acceptors [14], and the PCE is over 10% for the PSCs of fullerenes and their derivatives [15]. However, the further development of fullerenes and their derivatives is limited by obvious defects, such as the high cost of manufacturing and purification, poor co-solubility with polymers, and weak absorption in visible and infrared bands. Some PSCs have overcome these shortcomings by using small molecules instead of fullerenes, which have displayed higher performance over fullerenes in comparative devices [16,17,18,19,20].

Non-fullerene small molecule electron acceptors have attracted increasing attention today because of their advantages, such as their easy synthesis, adjustable energy gaps, and high absorption coefficients [21,22,23,24,25,26,27,28,29,30,31,32,33]. Lin Y. et al. found that non-fullerene acceptors (NFAs) can be used as potential electron acceptors to replace fullerene acceptors (FAs) [21]. Li N. et al. found that in organic solar cells (OSCs) containing poly(3-hexylthiophen-2,5-diyl) (P3HT), the PCE could be increased from 2.8% to 6.05% by replacing the FAs phenyl-C_61_-butyric acid methyl ester (PCBM) by the NFAs (5*Z*,5′*Z*)-5,5′-(((4,4,9,9-tetraoctyl-4,9-dihydro-*s*-indaceno[1,2-*b*:5,6-b′]dithiophene-2,7-diyl)bis(benzo[*c*]-[1,2,5]thiadiazole-7,4-diyl))bis-(methanylylidene))bis(3-ethyl-2-thioxothiazolidin-4-one)(O-IDTBR) [22]. Some excellent non-fullerene small electron acceptors have been reported [17,18,19,20,21,22,23,24,25,26,27,28,29,30,31,32,33], such as naphthalene diimide (NDI), perylene diimide (PDI), aromatic cores, and diketopyrrolopyrrole (DPP). The highest PCE for non-fullerene OSCs as non-fullerene small molecule acceptors for fused-ring electron acceptors has been over 15% [33].

**Figure 1:**
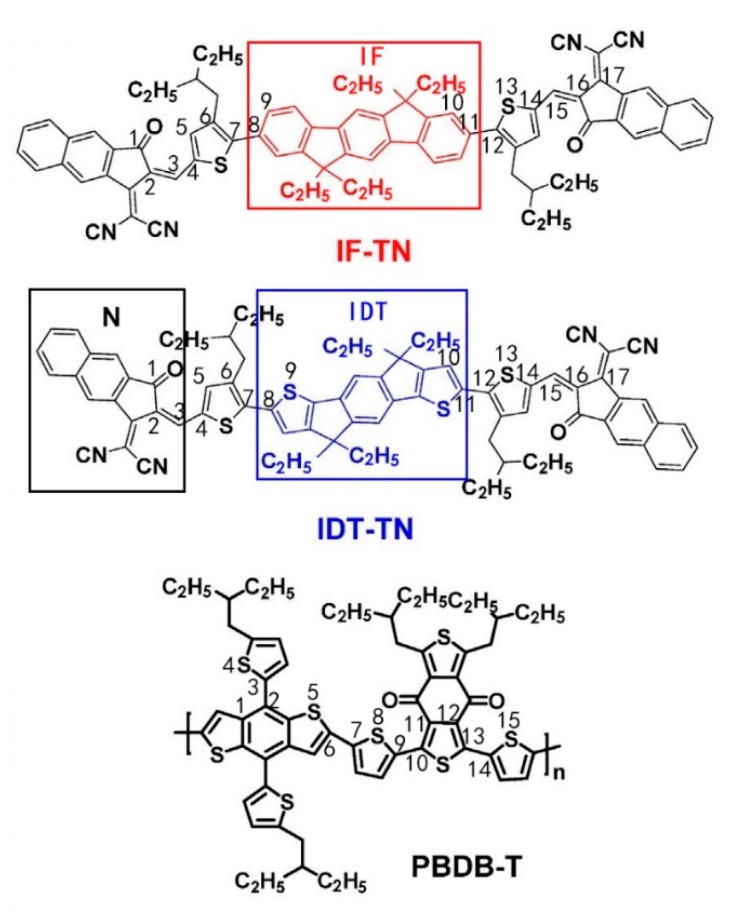
Molecular structure of the non-fullerene acceptors (NFAs) IF-TN, IDT-TN and donor PBDB-T.

Encouraged by a series of recent reports, we have investigated an electron-deficient acceptor unit, which is a novel moiety of 2-(3-oxo-2,3-dihydro-1H-cyclopenta [b]naphthalen-1-ylidene) malononitrile (N) connected to the end of the electron-donating core units. The electron-donating core units, indenofluorene (IF) and indacenodithiophene (IDT) can be used to form the NFAs IF-TN and IDT-TN (are shown in Figure 1) [34], where IDT can be made upon the replacement of the benzene ring in IF by thiophene. The geometric structures, and charge transport and photoelectric properties of the NFAs IF-TN, IDT-TN and donor polymer poly[(2,6-(4,8-bis(5-(2-ethylhexyl) thiophen-2-yl)benzo[1,2-*b*:4,5-*b*′]dithiophene)-co-(1,3-di(5-thiophene-2-yl)-5,7-bis(2-ethylhexyl)benzo[1,2-c:4,5-c′]dithiophene-4,8-dione)] (PBDB-T), and the charge transfer efficiencies at two interfaces PBDB-T/IF-TN (PB/IF) and PBDB-T/IDT-TN (PB/IDT) are studied by a quantum-chemical method (QM). The purpose of this work is to calculate and estimate the PCEs of two non-fullerene PSCs PBDB-T:IF-TN (PB:IF) and PBDB-T:IDT-TN (PB:IDT), by optimizing the geometrical structures of the NFAs IF-TN, IDT-TN, and PBDB-T, and constructing the interfaces at PB/IF and PB/IDT. We were able to obtain a series of photoelectric properties of the NFAs IF-TN and IDT-TN, and donor PBDB-T, such as HOMOs, LUMOs, ionization potentials (IPs), electron affinities (EAs), electrophilicity indexes (ω), electron and hole reorganization energies, absorption spectra, electron (hole) transport rates and mobilities, and the exciton separation rates at the PB/IF and PB/IDT interfaces. Open-circuit voltage (*V_OC_*), voltage loss (*V_loss_*), and fill factor (FF) are estimated by using molecular modeling.

## 2. Computational Methods

The ground state structures of the non-fullerene acceptors (NFAs) IF-TN and IDT-TN, and donor polymer PBDB-T (bond lengths, band angles, energy levels, ionization potentials (IPs), electron affinities (EAs), electrophilicity indexes, and reorganization energies) were optimized by using the density functional theory (DFT) [35] with B3LYP [36]/6-31G(d) method. The alkyl branches (R = $C_{4}H_{9}\&C_{8}H_{17}$) on the small molecule chains were substituted by ethyl groups to save computing resources (the alkyl branch of the polymer has no significant effect on the optoelectronic properties [37]). The optical parameters of the small molecules were investigated using the time-dependent DFT (TD-DFT) [38] with CAM-B3LYP [39]/6-31G(d) method. All calculations were performed by using Gaussian09 suite [40]. The parameters (exchange integral, reorganization energy, and free enthalpy of the reaction) of the D/A interfaces (PB/IF and PB/IDT) were all investigated using the DFT/B3LYP/6-31G(d) method involved in Marcus theory. The exchange integrals of the D/A interfaces both adopted the finite field method [41] and the Generalized Mulliken–Hush (GMH) [42] model. The charge difference density (CDD) plots of the small molecules IF-TN, IDT-TN, and PBDB-T, as well as the D/A interfaces were visualized by using Multiwfn3.6 [43]. The parameters (transfer integrals) of the NFAs (IF-TN and IDT-TN) were all investigated by using the DFT/PW91PW91/6-31G(d) method involved in Marcus theory [44].

## 3. Results and Discussion

### 3.1. Geometric Structures

The chemical structures (bond lengths and angles) of the NFAs (IF-TN and IDT-TN) are shown in Table 1 and Figure 1 (the bond lengths and angles of the donor PBDB-T are shown in Table S1). The bond length of C_7_–C_8_ is 1.446 Å in NFA IDT-TN, and the bond length of C_7_–C_8_ is 1.472 Å in NFA IF-TN. The bond angle of C_6_–C_7_–C_8_–S_9_ is –21.92° in NFA IDT-TN, and the bond angle of C_6_–C_7_–C_8_–C_9_ is –50.72° in NFA IF-TN. It can be seen that when the benzene ring in the electron-donating IF core unit (the red box portion in Figure 1) of NFA IF-TN is replaced by thiophene to obtain the IDT (the blue box portion in Figure 1) of NFA IDT-TN, the bond lengths and the absolute values of the bond angles between thiophene and thiophene are decreased significantly compared to those between thiophene and a benzene ring, which is due to the intermolecular repulsion between thiophene and thiophene. Improvement of the planarity of NFAs can effectively narrow the energy gap and broaden the optical absorption range of NFAs, which also effectively enhance the electron mobility of NFAs [45]. Furthermore, the donor polymer PBDB-T has a little degree of distortion (the largest dihedral angle is −56.31°).

At the same time, the aromaticity of IDT-TN has also been reduced upon substitution. Since the ionization potential (IP) of thiophene is lower than that of the benzene ring, the IP of NFA IDT-TN upon substitution has also been decreased. This can promote the separation of electrons from NFA IDT-TN, and when considering the relationship between the HOMO and the IP, the smaller energy gap of IDT-TN can be found. 

**Figure 2:**
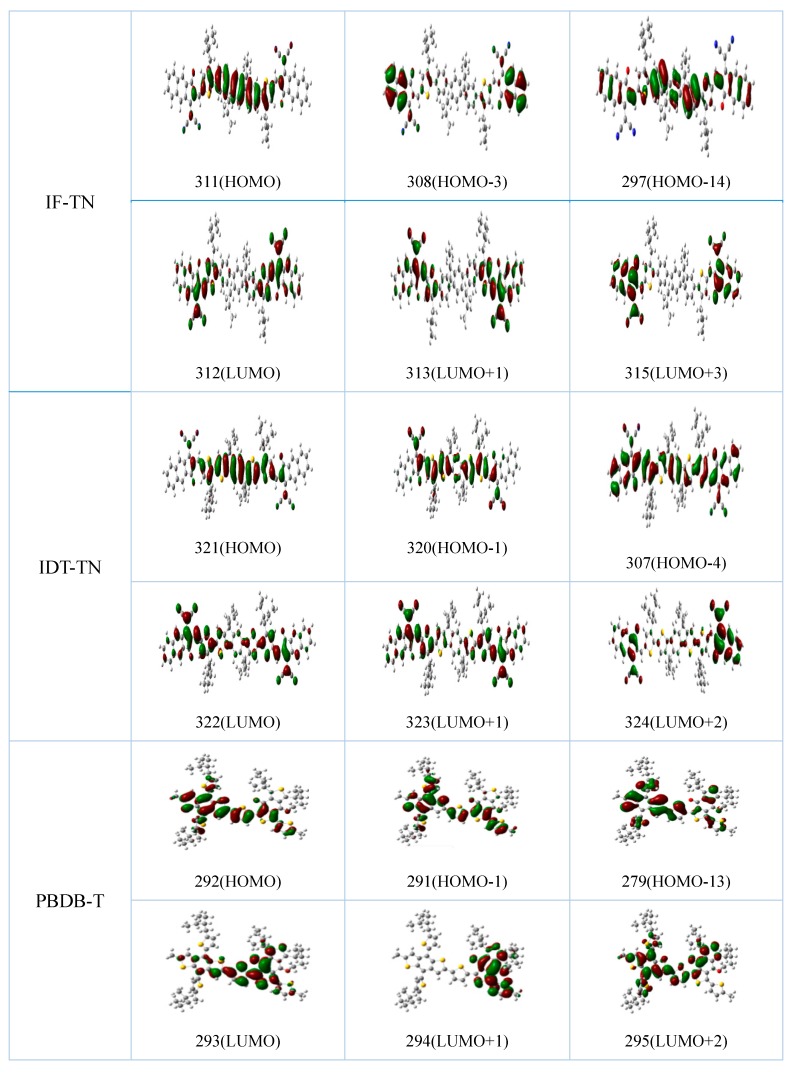
The frontier molecular orbital plots of the NFAs IF-TN and IDT-TN, and donor PBDB-T (where the red color represents electrons and green represents holes).

The energy gaps, energy levels, and frontier molecular orbitals (FMOs) of the NFAs (IF-TN and IDT-TN) and donor polymer PBDB-T are shown in Figure 2 and Table S2. It can be seen that the HOMO levels of the NFAs IF-TN and IDT-TN, and donor PBDB-T are −5.579, −5.251, and −4.932 eV, respectively, while the LUMO levels are −3.193, −3.299, and −2.302 eV, respectively. The energy gaps (∆_H-L_) are 2.386, 1.952, and 2.630 eV, respectively. It can be seen that the NFA IDT-TN has a lower energy gap than IF-TN, and the absorption peak of the NFA IDT-TN may have a wider absorption area and red-shift relative to IF-TN. Therefore, the non-fullerene PSC PB:IDT may have a higher J_SC_, owing to absorbing more solar energy [46].

It can be seen from Figure 2 that the electron clouds of the HOMOs of the NFAs IF-TN and IDT-TN are more concentrated near the IF (the red box portion in Figure 1) and IDT (the blue box portion in Figure 1) regions. The LUMOs are more concentrated near the N (the black box portion in Figure 1) regions. This describes that the IF (or IDT) and N have stronger electron-withdrawing abilities and hole-withdrawing abilities, respectively, compared to other parts of the NFAs. However, the IDT-TN has a stronger electron-withdrawing ability relative to IF-TN because of the structure of the electron-donating core units. Therefore, the electrons are transferred from the N to the IF (or IDT) region when the NFAs are stimulated by sunlight, and among the two systems, the IDT-TN is the better NFA because the IDT region can attract more electrons.

### 3.2. Ionization Potentials, Electron Affinities, and Reorganization Energies

Electron affinities (EAs) and ionization potentials (IPs) can be used to evaluate the degrees of difficulty of hole–electron binding and molecular ionization, respectively, which also represent the electron and hole barriers in the electron transport of the PSCs. Higher EAs and lower IPs can facilitate the injection of electrons and the separation of excitons, respectively [47,48]. The EAs and IPs of the NFAs IF-TN and IDT-TN, and donor PBDB-T are shown in Table 2, in which the EAs of the NFAs IF-TN and IDT-TN, and donor PBDB-T are 2.533, 2.666, and 1.482 eV, respectively, and the IPs are 6.272, 5.912, and 5.702 eV, respectively. The NFA IDT-TN has a higher EA and lower IP relative to IF-TN. Therefore, the PSC PB:IDT has a better efficiency of electron injection and exciton separation.

The electrophilicity index (ω) can be used to evaluate the degree of electrophilic power. The results show that lower hardness (ƞ) can lead to lower intramolecular charge transfer resistance, and that higher ω and electro-accepting power (ω^+^) can lead to stronger electron-withdrawing ability [49].

The *ƞ* values of the NFAs IF-TN and IDT-TN, and donor PBDB-T are 1.870, 1.623, and 2.110 eV, respectively, while the ω values are 5.184, 5.667, and 3.057 eV, respectively. The ω^+^ values are 3.216, 3.726, and 1.525 eV, respectively. One can know that the NFA IDT-TN has the lower *ƞ*, and higher ω and ω^+^, showing that IDT-TN should have lower intramolecular charge transfer resistance and stronger electron-withdrawing power compared to IF-TN.

The reorganization energy (λ) can be used to evaluate the degree of charge transport characteristics of PSCs, and lower hole-reorganization energy (λ_h_) can lead to a higher charge transfer rate [50,51,52,53,54]. The λ_h_ values of the NFAs IF-TN and IDT-TN, and donor PBDB-T are 0.196, 0.214, and 0.247 eV, respectively, while the λ_e_ values are 0.0906, 0.136, and 0.266 eV, respectively. Thus, IDT-TN may have the lower electron transport rate and electron mobility relative to IF-TN because of the higher λ_e_.

### 3.3. Optical Absorption Properties

**Figure 3:**
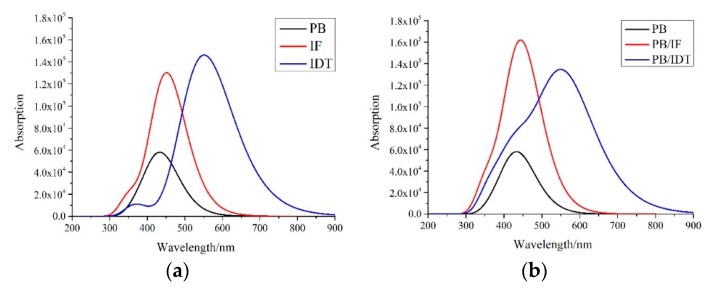
(**a**) The simulated absorption spectra of the NFAs IF-TN and IDT-TN, and donor PBDB-T. (**b**) The simulated absorption spectra of two interfaces (PB/IF and PB/IDT).

The active layer molecules absorbing solar energy are excited, causing them to produce excitons in the PSCs. The absorption spectra and excited-state lifetimes are important factors to measure the ability of an active layer to absorb solar energy. The transition energies, oscillator strengths (*f*), configuration interaction (CI) coefficients, and excited-state lifetimes (*τ*) for the NFAs IF-TN and IDT-TN, and donor PBDB-T are shown in Table 3 [55]. The absorption spectra and charge difference density (CDD) plots of the NFAs IF-TN and IDT-TN, and donor PBDB-T are shown in Figure 3a and Figure S1, respectively. At the same time, the transition energies, oscillator strengths, CI coefficients and light harvesting efficiency (LHE) of the interfaces PB/IF and PB/IDT are shown in Table S3 [56,57].

**Figure 4:**
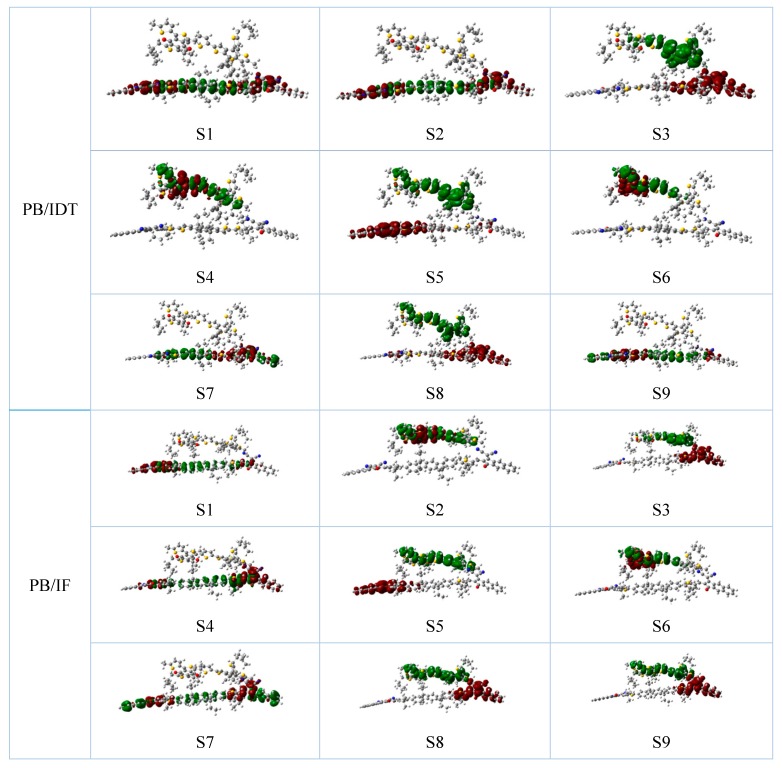
The representative charge difference density plots of two interfaces (PB/IF and PB/IDT) (red represents electrons, green represents holes).

The absorption spectra and charge difference density plots of the interfaces PB/IF and PB/IDT are shown in Figure 3b and Figure 4, respectively. The light absorption of the active layer is a key parameter of PSCs, that is to say, a good active layer should have broad and strong absorption spectra relative to the solar spectra. The absorption peaks of S1 of the NFAs IF-TN and IDT-TN, and donor PBDB-T are 452.24, 551.69, and 438.74 nm, respectively. The oscillator strengths are 3.1814, 3.5957, and 1.3271, respectively. The excited-state lifetimes are 0.964, 1.269, and 2.174 ns, respectively. 

As shown, for the NFAs IF-TN and IDT-TN, and donor PBDB-T, the strongest absorption state (where the oscillator strength *f* is largest) is the S1 excited state, which is the electron transfer from the HOMO to the LUMO level. The oscillator strength *f* of the S1 state and the excited-state lifetime *τ* are better for the NFA IDT-TN than for IF-TN. Therefore, IDT-TN has the obviously red-shifted absorption spectra and excitons staying in excited state for longer times. It can be seen from Figure 3a that the absorption peak of IDT-TN has an obvious red-shift relative to IF-TN, and IDT-TN also has the broader absorption spectra. The absorption peaks of S1 of the interfaces PB/IF and PB/IDT are 456.23 and 558.31 nm, respectively. The oscillator strengths are 2.1406 and 3.1576, respectively. The LHEs are 0.993 and 0.999, respectively. For the interfaces PB/IF and PB/IDT, the strongest absorption state is also the S1 excited state. Nevertheless, the electron transfers are from the HOMO-2 to LUMO levels or HOMO-1 to LUMO levels, respectively. The interface PB/IF has the larger oscillator strength *f* of the S1 state and LHE compared to PB/IF. It can be seen from Figure 3b that the interface PB/IDT has the obviously red-shifted absorption peak compared to PB/IF, and the PB/IDT also has the wider and stronger absorption spectra.

The charge difference densities (CDD) are analyzed and calculated in order to make the electron transfer processes of the NFAs and between donors and acceptors clearer and more intuitive [58]. As shown in Figure S1, for the NFAs IF-TN and IDT-TN, and donor PBDB-T, it can be seen that there are more electrons localized on the structures of electron-donating IDT core units of the NFA IDT-TN. For the interface PB/IF, it can be seen from Figure 4 that for the S1, S4, and S7 states, all electrons and holes are located on the NFA IF-TN. For the S2 and S6 states, all electrons and holes are located on the donor PBDB-T, while for the S3, S5, S8, and S9 states, electrons and holes are completely separated and distributed on the NFA and the donor, respectively. The S3 state is the minimum charge state, and the charges are completely separated at the PB/IF interface. When all electrons are located on the NFA IF-TN, the electrons are mainly located on the structures of electron-donating IF core units. For the PB/IDT interfaces, it can also be seen from Figure 4 that for the S1, S2, S7, and S9 states, all electrons and holes are located on the NFA IDT-TN. For the S4 and S6 states, all electrons and holes are located on the donor PBDB-T, while for the S3, S5, and S8 states, electrons and holes are completely separated and distributed on the NFA and the donor, respectively. The S3 state is viewed as the minimum charge state, and the charges are completely separated at the PB/IDT interface. When all electrons are located on the NFA IDT-TN, the electrons are mainly located on the structures of electron-donating IDT core units. Thus, it can be seen that the strongest absorption states are the S1 excited states for both PB:IF and PB:IDT active layers, and that the NFAs play key roles in absorbing sunlight.

### 3.4. The Excitons Separation Rate at the D/A Interfaces in Marcus Theory

After excitons are generated in the active layer, they diffuse to the D/A interfaces and separate to form free electrons and hole carriers at the D/A interfaces [59,60,61]. The excitons separation rate is an important factor to measure the efficiency of excitons separation at the D/A interfaces. The Marcus theory is used to estimate the excitons separation rate at D/A interfaces [44,62]:

(1) $$k_{CT}=\frac{{\left|V_{DA}\right|}^{2}}{\hbar }\sqrt{\frac{\pi }{\lambda k_{B}T}}\exp(\frac{-{\left(\Delta G_{CT}+\lambda \right)}^{2}}{4\lambda k_{B}T})$$

where *k_CT_*, *V_DA_*, *h*, *λ*, *k_B_*, *T*, and Δ*G_CT_* are represented as the excitons separation rate, charge transfer integration [16,41,63,64,65] (i.e., difference of the electrons coupling matrix element between the initial and final states), the Planck constant, reorganization energy [66,67,68], the Boltzmann constant, temperature (we supposed that *T* is 300 K at room temperature), and free energy change of the excitons [69] separation reaction, respectively, and the above calculation process is listed in the supporting materials. *V_DA_* can be calculated from Equations (S8) and (S9), *λ* can be calculated from Equations (S10) and (S11), and Δ*G_CT_* can be calculated from Equation (S12).

From Equation (S12), the exciton binding energies (*E_b_*) play an important role in the charge separation at the D/A interfaces in the PSCs, and the exciton binding energies (*E_b_*) approximately equal to the coulomb interaction energy (*E_coul_*) between the donor polymer and the NFAs during charge transfer [12,16,68]. The *E_b_* is also approximately equal to the difference between the optical band gap and the electrochemical band gap (the electrochemical band gap can be the difference between the HOMO and LUMO levels of the D/A interfaces, and the value is generally between 0.2 and 1 eV) [12,16,65]:

(2) $$E_{b}=\left|HOMO-LUMO\right|-E_{opt}$$

where HOMO, LUMO, and *E_opt_* refer to the HOMO of the interfaces, the LUMO of the interfaces, and the optical band gap that can be approximated as the first excited energy of the donor polymer.

**Figure 5:**
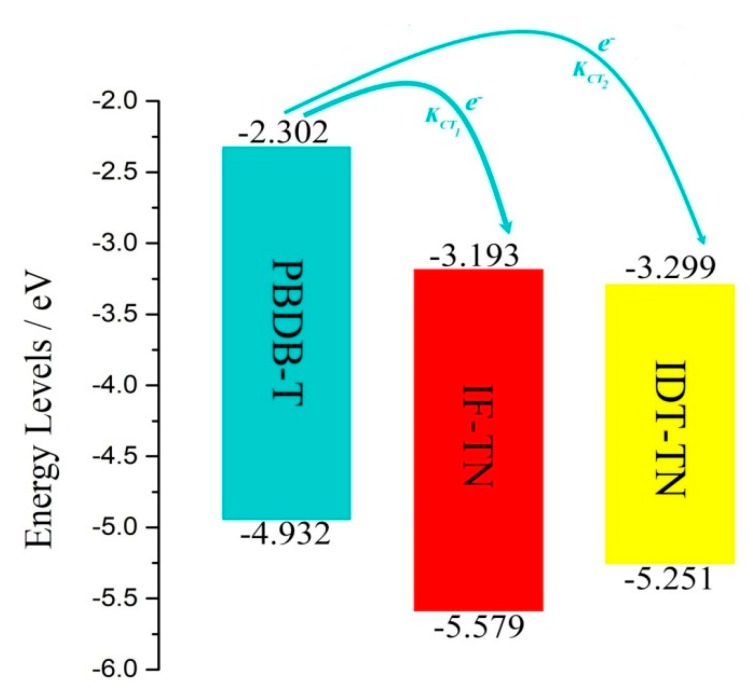
Energy levels of the NFAs IF-TN and IDT-TN and donor PBDB-T, and the charge transfer at the two interfaces (PB/IF and PB/IDT).

In the PSCs, the exciton binding energy and recombination energy should be as small as possible, and the charge transfer integration should be as large as possible. Under these conditions, the exciton separation rate can be as large as possible, meaning that the efficiency of producing the free electrons and hole carriers is further improved when the charge separation occurs at D/A interfaces. The *E_b_*, *V_DA_*, *λ*, Δ*G_CT_*, and *k_CT_* are shown in Table 4, and the k_CT1_ and k_CT2_ of the D/A interfaces PB/IF and PB/IDT can be seen in Figure 5.

It can be seen that the *E_b_* values of the interfaces PB/IF and PB/IDT are 0.970 and 0.631 eV, respectively. The *V_DA_* values are 0.316 and 0.184 eV, respectively, and the *λ* values are 0.478 and 0.505 eV, respectively. The Δ*G_CT_* values are −0.628 and −0.421 eV, respectively, while the *k_CT_* values of the interfaces are 1.538 × 10^15^ and 6.972 × 10^14^ s^−1^, respectively. As shown, the interface PB/IDT has a relatively smaller *k_CT_* relative to PB/IF because of the lower *V_DA_* and larger *λ*. At the same time, it can also be found that the interface PB/IDT has a larger difference between the LUMO of the donor polymer and the LUMO of IDT-TN (L_D_–L_A_), because IDT-TN has the lower LUMO (as can be seen in Figure 5). Furthermore, the D/A interface PB/IDT has the lower *E_b_* value, which has positive influence on the behavior of the D/A interface.

### 3.5. Electron Transport Rate and Mobility of NFAs 

After the excitons are separated at the interface to produce free electrons and hole carriers, the free electrons and hole carriers diffuse to the NFAs phase and donor polymer phase, respectively [45]. The electron transport rate and mobility are the key factors to measure the transport efficiency of free electrons in the NFAs phase. Higher electron transport rate and mobility of the NFAs can lead to having higher electron transport efficiency, and the PSCs can also have a higher short-circuit current (J_SC_). The transport of electrons in organic semiconductors is usually considered to be an incoherent transition process under the weak interaction of molecules at room temperature (T = 300 K), and in the discontinuous transition process, electrons are usually considered to migrate through the NFAs by jumping between adjacent molecules. Marcus theory is usually used to estimate the electron mobility of the NFAs when the temperature is high enough [44,62]:

(3) $$k=\frac{t^{2}}{\hbar }\sqrt{\frac{\pi }{\lambda k_{B}T}}\exp(\frac{-\lambda^{2}}{4k_{B}T})$$

where *λ*, *t*, *T*, and *k_B_* represent the reorganization energy, charge transfer integral, room temperature (300 K), and the Boltzmann constant, respectively. According to Kupman’s theorem, in the electron transport reaction of an anionic system (electron transport) and the electron transport reaction of a cationic system (hole transport), the charge transfer integrals can be approximately considered as half of the difference of energy between the LUMO + 1 and LUMO of two adjacent neutral systems [70,71]:

(4) $$t_{e}=\frac{E_{LUMO+1}-E_{LUMO}}{2}$$

As the acceptor materials of the active layer in the PSCs (electron transport layer), the NFAs should have good electron transport ability, and as the donor materials of the active layer in the PSCs (hole transport layer), the donor polymers should have good hole transport ability. A high electron transport rate of the NFAs should lead to high electron mobility, which indicates that the NFAs should have high electron transport ability and should have great potential as good electron transport materials.

According to the Einstein equation, electron mobility can be calculated as [72,73]:

(5) $$\mu =\frac{e}{k_{B}T}D$$

where *k_B_* represents the Boltzmann constant, *T* represents room temperature (300 K), *e* represents electron charge, and *D* represents the diffusion coefficient. When electrons are migrated by means of jumping along with a particular path (one-dimensional), electron mobility can be calculated as [73,74,75]:

(6) $$\mu =\frac{er^{2}}{2k_{B}T}k$$

where *r* and *K* are the jumping distance between adjacent molecules and the charge transfer rate, respectively. The electron transfer integral *t_e_*, electron reorganization energy *λ_e_*, electron transport rate *k_e_*, electron diffusion constant *D_e_*, and the electron mobility *µ_e_* of the NFAs IF-TN and IDT-TN are shown in Table 5. The hole transfer integral *t_h_*, hole reorganization energy *λ_h_*, hole transport rate *k_h_*, hole diffusion constant *D_h_*, and the hole mobility *µ_h_* of the NFAs IF-TN and IDT-TN can be seen in Table S4. 

As shown in Table 5, the electron transfer integral *t_e_*, electron reorganization energy *λ_e_*, electron transport rate *k_e_*, electron diffusion constant *D_e_*, and the electron mobility *µ_e_* of the NFA IF-TN are 0.00395 eV, 0.0906 eV, 3.607 × 10^12^ s^−1^, 4.299 × 10^−4^ cm^2^/s, and 0.0166 cm^2^/(V⋅s), respectively. The electron transfer integral *t_e_*, electron reorganization energy *λ_e_*, electron transport rate *k_e_*, electron diffusion constant *D_e_*, and electron mobility *µ_e_* of the NFA IDT-TN are 0.00898 eV, 0.136 eV, 9.822 × 10^11^ s^−1^, 1.145 × 10^−3^ cm^2^/s, and 0.0443 cm^2^/(V⋅s), respectively. It can be seen that the NFA IDT-TN has a higher *µ_e_* because of the larger *t_e_*. The value of *µ_e_* for the NFA IDT-TN is about three times that of the NFA IF-TN, which means that IDT-TN is the better electron transport material compared to IF-TN.

According to the structures of the NFAs IF-TN and IDT-TN, one of the reasons why the NFA IDT-TN has the larger electron mobility (*µ_e_*) is the fact that the aromaticity of IDT-TN is reduced, and the electron transfer integral is increased, which promotes the free movement of electrons intermolecular. The PSC PB:IDT may have a higher short-circuit current (J_SC_).

### 3.6. Macroscopic Properties of Non-Fullerene PSCs

It is well known that the open-circuit voltage (*V*_OC_), fill factor (FF), and power conversion efficiency (PCE) are all very important parameters for evaluating the macroscopic properties of non-fullerene PSCs. The *V*_OC_ values of the PSCs can be approximately considered to be in direct proportion to the absolute values of the differences between HOMOs of donor polymer and LUMOs of the NFAs [76], and the NFAs with the higher LUMO levels have higher *V*_OC_ values. In theory, Equation (7) is used to estimate the *V*_OC_ of the PSCs [77].

(7) $$V_{OC}=\frac{1}{e}(E_{HOMO}(D)-E_{LUMO}\left(A\right))-\Delta V$$

where *E*_HOMO_ (D), *E*_LUMO_ (A), e, and ∆*V* are represented as the HOMO level of the donor polymer, the LUMO levels of the NFAs, the elemental charge, and the empirical constant (usually considered to be 0.3 *V* [78]), respectively. The fill factor (FF) is an important parameter for describing the photoelectric properties of the PSCs and calculating the PCE, and the fill factor in an ideal state FF_0_ (ignoring the effects of parallel resistance and series resistance) can be calculated as [79,80]:

(8) $$FF_{0}=\frac{\nu_{oc}-\ln(\nu_{oc}+0.72)}{\nu_{oc}+1}$$

where the *V*_OC_ (*V*_OC_ is normalized to the thermal voltage) can be expressed by Equation (S15) [79,80], and the voltage loss (*V*_loss_) can be expressed by Equation (S16) [81]. The energy gaps ($E_{g}$) of the donor, optical bandgaps (*E_g_*) of the active layers, differences between the LUMOs of donor polymers and the LUMOs of the NFAs (L_D_–L_A_), *V*_OC_, *V*_loss_, and the fill factors in the ideal state (FF_0_) of the PSCs PB:IF and PB:IDT are shown in Table 6. 

The $E_{g}$, *E_g_*, L_D_–L_A_, *V*_OC_, *V*_loss_, and FF_0_ values of the PSC PB:IF are 2.631 eV, 2.718 eV, 0.563 eV, 1.439 V, 1.279 V, and 91.12%, respectively. The $E_{g}$, *E_g_*, L_D_–L_A_, *V*_OC_, *V*_loss_, and FF_0_ values of the PSC PB:IDT are 2.631 eV, 2.221 eV, 0.669 eV, 1.333 V, 0.888 V, and 90.57%, respectively. As shown, the PSC PB:IDT has the relatively smaller *V*_OC_ and FF_0_, but the differences of the *V*_OC_ and FF_0_ values between the two NFAs are little. The PSC PB:IDT has a significantly lower *V*_loss_, which may lead to improvement of the *V*_OC_ quality. The PSC PB:IDT also has a significantly higher µ_e_, which may lead to improvement of the FF_0_ [82].

**Figure 6:**
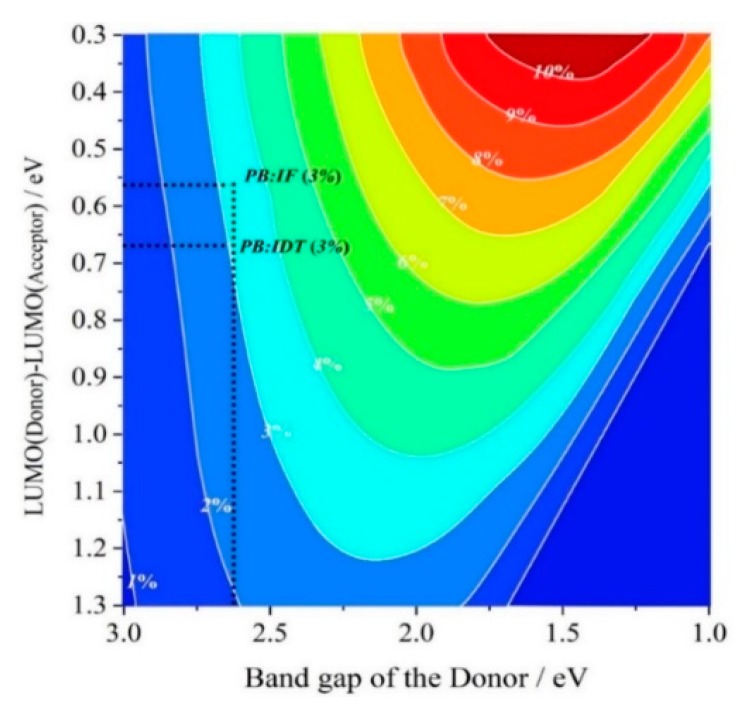
Predicted power conversion efficiency (PCE) for the PSCs PB:IF and PB:IDT with the Scharber diagram.

For the power conversion efficiency (PCE), the Scharber diagram (by the contour lines and colors) is used to estimate the specific value of the PCE [83,84]. The Scharber diagram can be seen in Figure 6, and the PCE values of the PSCs PB:IF and PB:IDT are both 3%. Although the PSC PB:IF may have a higher *V*_OC_ and FF_0_, the PSC PB:IDT has the wider, stronger, and more red-shifted absorption spectra, larger LHE, longer excited-state lifetime, higher voltage loss, and larger electron mobility. It is hoped that on the same order of magnitude for *V*_OC_ and FF_0_, the PSC based on PB:IDT has the bigger external quantum efficiency (EQE) and short-circuit current (J_SC_), which should lead to a better PCE. 

### 3.7. Built-In Electric Field Effect on the Optical Character

Built-in electric fields have effects on the optical properties of the donor polymers and NFAs by affecting the internal photochemistry of the PSCs [85,86]. The optical properties of the PSC PB:IDT under different built-in electric fields are calculated and analyzed to study the effects of built-in electric fields on the optical properties of PSC PB:IDT. The optical characters and absorption spectra of PSC PB:IDT under different built-in electric fields can be seen in Table 7 and Figure S2, respectively. The oscillators of the S1 state (where the oscillator strength *f* is the largest) of PSC PB:IDT are 3.1576, 3.1344, 3.0767, and 3.0007, respectively, and the absorption peaks are at 558.31, 558.48, 559.76, and 561.95 nm, respectively.

Under the electric fields of 0, 10 × 10^−4^, 20 × 10^−4^, and 30 × 10^−4^ a.u., the absorption spectra of the PSC PB:IDT have gradually red-shifted (red-shift increments were 0.17, 1.45, and 3.64 nm under the built-in electric fields of energy intensity 0, 10 × 10^−4^, 20 × 10^−4^, and 30 × 10^−4^, respectively, relative to the PSC PB:IDT without any built-in electric field), and the gene-base for oscillator strengths is increased. This result illustrates that the built-in electric fields have significant effects on the photoelectric properties of the PSC PB:IDT, and the appropriate increase of built-in electric fields may be helpful to improve the optical absorption effects of the PSC PB:IDT.

## 4. Conclusions

In this paper, the photoelectric performances of two non-fullerene PSCs were calculated to investigate and compare the two NFAs’ (IF-TN and IDT-TN) abilities by the density functional theory (DFT) and time-dependent density functional theory (TD-DFT). At the same time, the structures and properties of the D/A interfaces PB/IF and PB/IDT were simulated by a quantum-chemical method (QM) and Marcus theory. The results show that: (a) For the NFAs IF-TN and IDT-TN, the NFA IDT-TN has the narrower energy gap, lower IPs, higher EAs, a larger electrophilicity index ω, a longer excited-state lifetime τ, larger absorption peaks, better planarity, a greater electron transport rate *k_e_*, and greater mobility *µ_e_*; (b) for the D/A interfaces PB/IF and PB/IDT, the D/A interface PB/IDT has the stronger and more red-shifted absorption spectra, bigger LHE, a smaller exciton binding energy *E_b_*, and a slightly smaller excitons separation rate with little difference in value compared to that of PB/IF; (c) for the macro-photoelectric performance of non-fullerene PSCs (PB:IF and PB:IDT), the non-fullerene PSC PB:IDT has the lower voltage loss *V*_loss_, and the slightly smaller open-circuit voltage *V*_OC_ and fill factor FF_0_ with little differences in values compared to those of PB:IF, but however, the Jsc for the PB:IDT system has been significantly increased by substitution. It can be predicted that the PCEs of the non-fullerene PSCs (PB:IF and PB:IDT) can both reach 3% (and the PB:IDT should have a higher PCE). Finally, we can infer that the non-fullerene PSC PB:IDT should have more potential applications in solar cells.

## Figures and Tables

**Table 1:** The bond lengths and bond angles for non-fullerene acceptor-based IF-TN and IDT-TN.

Length (Å)	C_2_-C_3_	C_3_-C_4_	C_7_-C_8_	C_11_-C_12_	C_14_-C_15_	C_15_-C_16_
IF-TN	1.380	1.425	1.472	1.471	1.424	1.380
IDT-TN	1.381	1.422	1.446	1.442	1.421	1.382
Angle (°)	C_1_-C_2_-C_3_-C_4_	C_2_-C_3_-C_4_-C_5_	C_6_-C_7_-C_8_-C(S)_9_	C_10_-C_11_-C_12_-S_13_	C_13_-C_14_-C_15_-C_16_	C_14_-C_15_-C_16_-C_17_
IF-TN	3.81	4.49	-50.72	43.79	176.38	−179.88
IDT-TN	2.95	3.49	−21.92	26.94	175.31	−179.36

**Table 2:** Ionization potentials, electron affinities, hardness, electrophilicity indexes, electro-accepting powers, and reorganization energies of the NFAs IF-TN and IDT-TN, and donor PBDB-T.

*E* (eV)	IF-TN	IDT-TN	PBDB-T
IP	6.272	5.912	5.702
EA	2.533	2.666	1.482
*ƞ*	1.870	1.623	2.110
ω	5.184	5.667	3.057
ω^+^	3.216	3.726	1.525
λ_h_	0.196	0.214	0.247
λ_e_	0.0906	0.136	0.266

**Table 3:** Values for transition energy *E* (eV), absorption peak λ (nm), oscillator strength, configuration interaction (CI) coefficient and excited-state lifetime *τ* (ns) of the NFAs IF-TN, IDT-TN and donor PBDB-T.

Molecules	State	*E*	λ	Contribution MOs ^[a]^	Strength *f*	*τ*
IF-TN	S1	2.7416	452.24	H→L (0.49617)	3.1814	0.964
	S2	2.8836	429.97	H→L+1 (0.45564)	0.0171	
	S3	3.4787	356.41	H-3→L+1 (0.38543)	0.4632	
	S4	3.4953	354.71	H-3→L (0.38995)	0.0084	
	S5	3.5148	352.75	H-14→L+3 (0.31064)	0.0278	
	S6	3.5173	352.50	H-13→L+1 (0.28640)	0.0141	
IDT-TN	S1	2.2474	551.69	H→L (0.58699)	3.5957	1.269
	S2	2.5793	480.69	H→L+1 (0.54405)	0.0258	
	S3	3.3436	370.81	H-1→L+1 (0.36306)	0.2604	
	S4	3.3676	368.17	H-1→L (0.36734)	0.0062	
	S5	3.4431	360.09	H→L+2 (0.36588)	0.0036	
	S6	3.4958	354.67	H-14→L+2 (0.25121)	0.0010	
PBDB-T	S1	2.8259	438.74	H→L (0.61234)	1.3271	2.174
	S2	3.2606	380.25	H→L+1 (0.48889)	0.3204	
	S3	3.5243	351.79	H-1→L (0.51487)	0.1427	
	S4	3.6137	343.10	H-14→L (0.23288)	0.0067	
	S5	3.6200	342.50	H-13→L+1 (0.45320)	0.0108	
	S6	3.8745	320.00	H→L+2 (0.46964)	0.1878	

[a]: H and L represent HOMO and LUMO, respectively.

**Table 4:** Calculated exciton binding energy *E_b_* (eV), charge transfer integral *V_DA_* (eV), reorganization energy *λ* (eV), Gibbs free energy change Δ*G_CT_* (eV) and excitons separation rate *k_CT_* (s^−1^) of two interfaces (PB/IF and PB/IDT).

Interfaces	*E_b_*	*V_DA_*	*λ*	Δ*G_CT_*	*k_CT_*
PB/IDT	0.631	0.184	0.505	−0.421	$6.972\times 10^{14}$
PB/IF	0.970	0.316	0.478	−0.628	$1.538\times 10^{15}$

**Table 5:** Calculated electron transfer integral *t_e_* (eV), electron reorganization energy *λ_e_* (eV), electron transport rate *k_e_* (s^−1^), distance *r* (Å), electron diffusion constant *D_e_* ($cm^{2}∕s$), and the electron mobility *µ_e_* ($cm^{2}∕\left(V\cdot s\right)$) of the NFAs IF-TN and IDT-TN.

Dimers	*t_e_*	*λ_e_*	*k_e_*	*r*	*D_e_*	*μ_e_*
IF-TN	0.00395	0.0906	3.607 × 10^12^	4.8824	4.299 × 10^−4^	0.0166
IDT-TN	0.00898	0.136	9.822 × 10^11^	4.8287	1.145 × 10^−3^	0.0443

**Table 6:** Calculated energy gaps $E_{g}$ of donor (eV), optical bandgap *E_g_* of the active layer (eV), difference between the HOMO of the donor and the LUMO of the acceptor H_D_–L_A_ (eV), difference between the LUMO of the donor and the LUMO of the acceptor L_D_–L_A_ (eV), the open-circuit voltage $V_{OC}$ (V), voltage loss $V_{loss}$ (V), and fill factor in the ideal state FF_0_ (%) of two polymer solar cells (PSCs) (PB:IF and PB:IDT).

Cells	$E_{g}$	*E_g_*	H_D_-L_A_	L_D_-L_A_	*V_OC_*	*V_loss_*	ν_oc_	FF_0_
PB:IF	2.631	2.718	1.739	0.563	1.439	1.279	55.663	91.12
PB:IDT	2.631	2.221	1.633	0.669	1.333	0.888	51.563	90.57

**Table 7:** Built-in electric field effects on optical character of the PSC PB:IDT.

Field (×10^−4^ a.u.)	Peak of S1/nm	Engrgy/eV	Contribution Mos ^[a]^	Strength *f*
0	558.31	2.221	H-1→L (0.53767)	3.1576
10	558.48	2.220	H→L (0.50854)	3.1344
20	559.76	2.215	H→L (0.49961)	3.0767
30	561.95	2.206	H→L (0.49280)	3.0007

[a]: H and L represent HOMO and LUMO, respectively.

## Supplementary Materials

The following are available online at https://www.mdpi.com/2073-4360/11/6/958/s1.
