# Supplementary material for: Modification of NFA-Conjugated Bridges with Symmetric Structures for High-Efficiency Non-Fullerene PSCs
Source: Polymers (Basel). 2019 Jun 2;11(6):958. doi: 10.3390/polym11060958 (PMC6630734; doi:10.3390/polym11060958)
Supplement: Supplementary file 1 [file polymers-11-00958-s001.pdf]

Modification of NFA Conjugated-Bridges with Symmetric Structures for High-Efficiency Non-Fullerene PSCs

Qiuchen Lu [1], Ming Qiu [1], Meiyu Zhao [2,*], Zhuo Li [1] and Yuanzuo Li [1,*]

[1] College of Science, Northeast Forestry University, Harbin 150040, China; qiuchenlu1997@126.com (Q.L.); qm15765526850@163.com (M.Q.); 17733720476@163.com (Z.L.)
[2] School of Chemistry and Chemical Engineering, Harbin Institute of Technology, Harbin 150001, China
* Correspondence: myzhao@hit.edu.cn (M.Z.); yzli@nefu.edu.cn (Y.L.); Tel.: +86-451-8640-3305 (M.Z.); +86-451-8219-2245-8211 (Y.L.)

Table S1. bond length and band angle for the donor PBDB-T

Length (Å)	C_2-C_3	1.474	Angle (°)	C_1-C_2-C_3-S_4	-56.31
	C_6-C_7	1.448		S_5-C_6-C_7-S_8	23.06
	C_9-C_{10}	1.449		S_8-C_9-C_{10}-C_{11}	10.07
	C_{13}-C_{14}	1.451		C_{12}-C_{13}-C_{14}-S_{15}	20.72

Table S2. Energy levels and band gaps for Non-Fullerene Acceptor-Based IF-TN, IDT-TN and donor PBDB-T

[a]:H and L represent HOMO and LUMO, respectively

E/eV	H-2[a]	H-1	H	L	L+1	L+2	Δ_{H-L}
IF-TN	-6.260	-6.043	-5.579	-3.193	-3.150	-2.396	2.386
IDT-TN	-6.275	-5.855	-5.251	-3.299	-3.158	-2.423	1.952
PBDB-T	-5.852	-5.318	-4.932	-2.302	-2.143	-1.467	2.630

Table S3. Transition energy, absorption peak, oscillator strength, LHE and CI coefficient for two interfaces

Interfaces	state	E(eV)	λ(nm)	Contribution MO[a]	Strength *f*	LHE(λ)
PB/IDT	S1	2.2207	558.31	H-1→L (0.53767)	3.1576	0.999
	S2	2.5406	488.01	H-1→L+1 (0.51778)	0.0018	
	S3	2.7001	459.19	H→L (0.64757)	0.0074	
	S4	2.8332	437.61	H→L+3 (0.59442)	1.4936	
	S5	3.1627	392.02	H→L+1 (0.64854)	0.0000	
	S6	3.2625	380.02	H→L+5 (0.44960)	0.2477	
PB/IF	S1	2.7176	456.23	H-2→L (0.38415)	2.1406	0.993
	S2	2.8403	436.52	H→L+2 (0.57211)	1.4498	
	S3	2.8670	432.45	H→L (0.59601)	0.0136	
	S4	2.8754	431.19	H-3→L (0.35418)	0.4111	
	S5	3.2807	377.92	H→L+1 (0.59764)	0.0001	
	S6	3.2890	376.97	H→L+5 (0.45117)	0.2848	

Table S4. Calculated hole transfer integral t_h (eV), hole reorganization energy λ_h (eV), hole transport rate k_h (s^{-1}), distance r (Å), hole diffuse constant D_h (cm^2 / s) and the hole mobility μ_h $(cm^2 / (V \cdot s))$ of the NFAs IF-TN and IDT-TN

Dimers	t_h	λ_h	k_h	r	D_h	μ_h
IF-TN	0.0191	0.196	2.064×10^{12}	4.8824	2.460×10^{-3}	0.0952
IDT-TN	0.0114	0.214	5.965×10^{11}	4.8287	6.954×10^{-4}	0.0269

Figure captures

Figure S1. The charge difference density plots of the NFAs IF-TN, IDT-TN and donor PBDB-T (where the cyan color represents electrons and violet represents holes)
Figure S2. The simulated absorption spectra of PB:IDT PSCs under different built-in electric field

Figure S1. The charge difference density plots of the NFAs IF-TN, IDT-TN and donor PBDB-T (where the cyan color represents electrons and violet represents holes)

states	IF-TN	IDT-TN	PBDB-T
S1	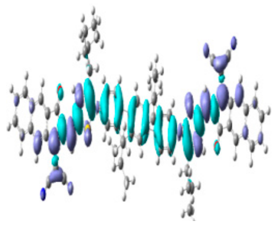	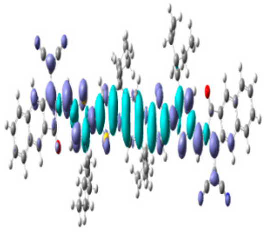	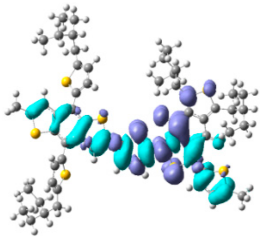
S2	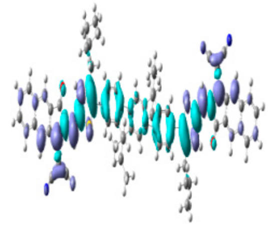	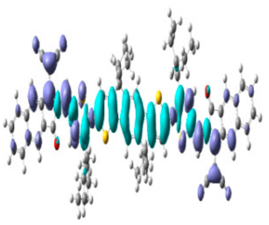	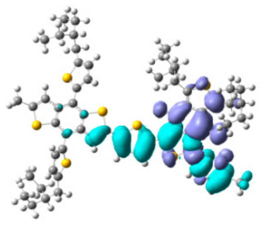
S3	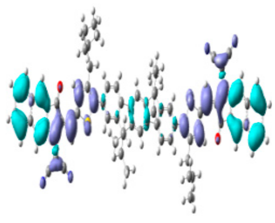	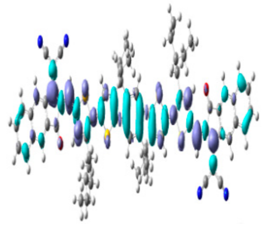	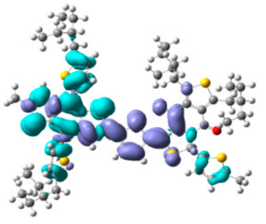
S4	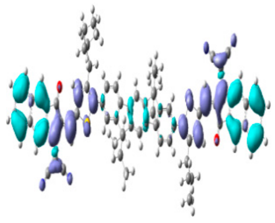	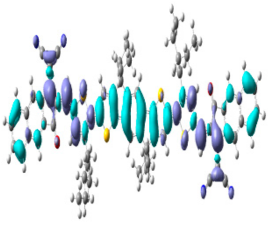	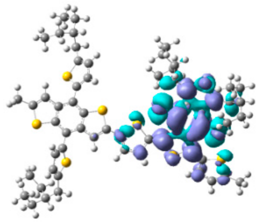
S5	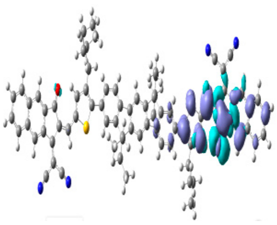	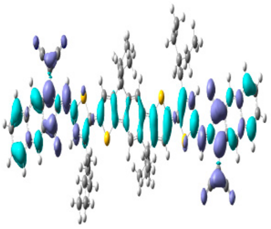	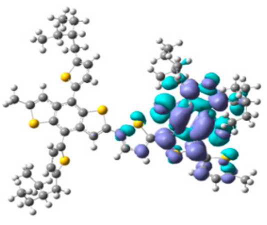
S6	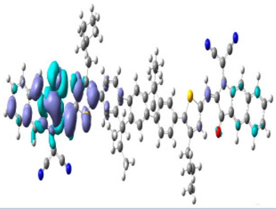	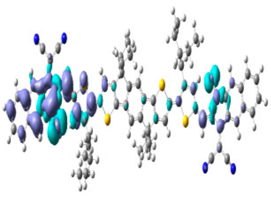	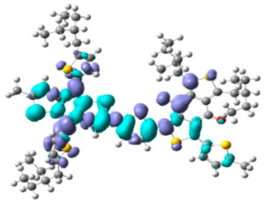

Figure S2. The simulated absorption spectra of PB:IDT PSCs under different built-in electric field

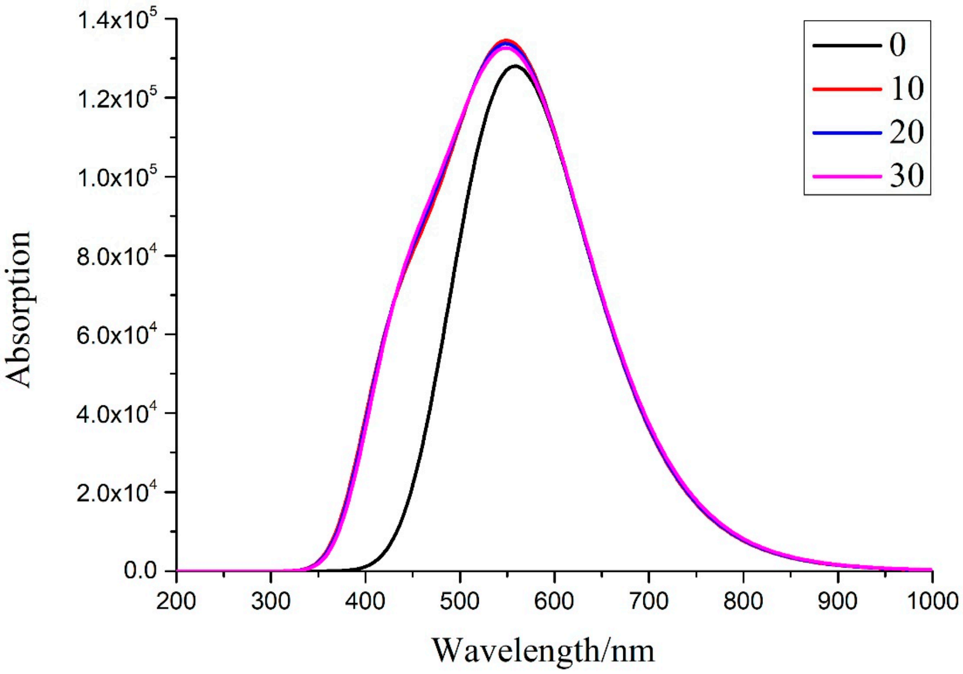

The η, ω and ω^+ can be calculated by the Eq. S1, Eq. S2 and Eq. S3, respectively [1]:

$$\eta = \frac{IP - EA}{2} \qquad \text{(S1)}$$

$$\omega = \frac{(IP + EA)^2}{4 \cdot (IP - EA)} \qquad \text{(S2)}$$

$$\omega^+ = \frac{(IP + 3 \cdot EA)^2}{16 \cdot (IP - EA)} \qquad \text{(S3)}$$

The hole reorganization energy λ_h and electron reorganization energy λ_e can be calculated by the Eq. S4 and Eq. S5, respectively [2-5]:

$$\lambda_h = (E_0^+ - E_+) + (E_+^0 - E_0) \qquad \text{(S4)}$$

$$\lambda_e = (E_0^- - E_-) + (E_-^0 - E_0) \qquad \text{(S5)}$$

Where E_0, E_+/E_-, E_0^+/E_0^- and E_+^0/E_-^0 can be calculated by the neutral molecule, cation (anion) under the ground state and the cation (anion), neutral molecule under the cationic (anionic) state, respectively.

The excited-state lifetime τ can be calculated by Eq. S6 [6]:

$$\tau = \frac{1.499}{fE^2} \qquad \text{(S6)}$$

where E and f are the excited-state energy (cm^{-1}) and the oscillator strength, respectively; The light harvesting efficiency (LHE) can be calculated by Eq. S7 [7, 8]:

$$LHE(\lambda) = 1 - 10^{-f} \qquad \text{(S7)}$$

The charge transfer integration (i.e. the difference between electrons coupling matrix element of initial state and final state) plays a key factor in the excitons separation rate at the D/A interfaces. By the GMH model, it can be calculated as [9, 10]:

$$V_{DA} = \frac{\mu_{tr} \Delta E}{\sqrt{(\Delta\mu)^2 + 4(\mu_{tr})^2}} \qquad \text{(S8)}$$

Where ΔE, μ_{tr} and Δμ represent the vertical excitation energy, transition dipole moment between two states (the orientation is from donor polymer to NFAs) and the difference of the dipole moments between the initial state and final state which can be calculated by the Hellmann-Feynman theorem, respectively; The difference of the dipole moments between the initial state and excited state Δμ can be calculated by the

finite field method, which can hinge on the equation represented by the electric field F [11-13]:

$$E_{ext}(F) = E_{ext}(0) - \Delta\mu F - \frac{1}{2}\Delta\alpha F^2 \qquad \text{(S9)}$$

Where $E_{ext}(0)$, Δμ and Δα are the lowest excitation energy of the charge transfer excited between the molecules in zero electric field status, a difference of the dipole moments between the initial state and final state, change rate of the polarizability, respectively.

The reorganization energy λ at D/A interface can be classified into the external recombination energy λ_s and internal reorganization energy λ_{in}. And the internal reorganization energy λ_{in} at D/A interfaces between charge minimum completely separated state and ground state can be calculated as [14, 15]:

$$\lambda_{in} = E(A^-) - E(A) + E(D) - E(D^+) \qquad \text{(S10)}$$

Where E(A) and $E(A^-)$ represent the energies of the optimal ground state of the NFAs and the NFAs of electrically neutral on anion structure, respectively; $E(D^+)$ and E(D) represent the energies of optimized donor polymer cation and donor polymer cation on electrically neutral structure, respectively; The external recombination energy λ_s can be approximately equal to the solvent reorganization energy λ_{ex} because the electron polarization caused by the overall geometric relaxation of molecules in a solvent may cause external recombination. So, it can be approximated as [16]:

$$\lambda_s \approx \lambda_{ex} = (\Delta q)^2 (\frac{1}{\varepsilon_{op}} - \frac{1}{\varepsilon_s})(\frac{1}{2r_1} + \frac{1}{2r_2} + \frac{1}{d}) \qquad \text{(S11)}$$

Where Δq, ε_{op}, ε_s, r_1 and r_2 are the transferred charge amount, optical permittivity of the solvent, electrical permittivity of the solvent, radius of the NFAs and donor polymer, respectively; The external recombination energies λ_s (the solvent reorganization energy λ_{ex}) are approximately equal to 0.29 eV because that the λ_s of the electron acceptors are hard to estimate in theory and some studies had shown that the λ_s of the electron acceptor in the polar solvent was circa 0.29 eV [16].

From the Rehm-Weller equation, the change of Gibbs free energy ΔG_{CT} of exciton separation reaction at the D/A interfaces can be expressed as [17]:

$$\Delta G_{CT} = EA(A) - IP(D) - \Delta E_{0-0} - E_b \qquad \text{(S12)}$$

Where EA(A), IP(D), E_b and E_{0-0} are represented as the EA of the NFAs, IP of the donor polymer, exciton binding energy at the D/A interfaces (coulomb interaction

energy between donor polymer and NFAs during charge transfer) and the lowest singlet excitation energy of the donor polymer, respectively.

The Coulomb interaction energy E_{coul} can be calculated as [11, 16, 18]:

$$E_{\mathrm{b}} \approx E_{\mathrm{coul}} = \frac{1}{4\pi\varepsilon_0\varepsilon_r}\sum_{i\in D^+}\sum_{j\in A^-}\frac{q_i q_j}{r_{ij}} = \frac{q_D q_A}{4\pi\varepsilon_0\varepsilon_r r_{DA}} \quad \text{(S13)}$$

Where ε_0, ε_r, q_D, q_A and r_{DA} refer to the vacuum dielectric constant, relative dielectric constant, charges of the donor polymer, charges of the NFAs, distance between donor polymer and NFAs, respectively.

The charge transfer integrals can be approximately considered as the half of the difference of energy between HOMO and HOMO-1 of two adjacent neutral systems [19, 20]:

$$t_h = \frac{E_{HOMO} - E_{HOMO-1}}{2} \quad \text{(S14)}$$

The ν_{oc} (the open-circuit voltage V_{OC} normalized to the thermal voltage) can be expressed as [21, 22]:

$$\nu_{oc} = \frac{eV_{oc}}{k_B T} \quad \text{(S15)}$$

Where k_B, T and e are represented as Boltzmann constant, room temperature (300 K) and elemental charge, respectively; And the voltage loss V_{loss} is also a key parameter to describe the photoelectric properties of the PSCs and evaluating the quality of open-circuit voltage V_{OC}, which can be calculated as [23]:

$$V_{loss} = \frac{E_g}{q} - V_{OC} \quad \text{(S16)}$$

Where q, E_g and E_g present elemental charge, the energy gaps of donor polymer and the optical bandgap of active layer in PSCs, respectively.

References:

1. Chattaraj, P. K.; Sarkar, U.; Roy, D. R., *Chem. Rev.* **2006,** *106*, 2065-2091.

2. Zou, L. Y.; Ren, A. M.; Feng, J. K.; Liu, Y. L.; Ran, X. Q.; Sun, C. C., *J. Phys. Chem. A* **2008,** *112*, 12172-12178.

3. Kose, M. E.; Mitchell, W. J.; Kopidakis, N.; Chang, C. H.; Shaheen, S. E.; Kim, K.; Rumbles, G., *J. Am. Chem. Soc.* **2007,** *129*, 14257-14270.

4. Janprapa, N.; Vchirawongkwin, V.; Kritayakornupong, C., *Chem. Phys.* **2018,** *510*, 60-69.

5. Shi, X.; Yang, Y.; Wang, L.; Li, Y., *J. Phys. Chem. C* **2019**, *123*, 4007-4021.

6. Ren, P.; Sun, C.; Shi, Y.; Song, P.; Yang, Y.; Li, Y., *J. Mater. Chem. C* **2019**, *7*, 1934-1947.

7. Ardo, S.; Meyer, G. J., *Chem. Soc. Rev.* **2009,** *38*, 115-164.

8. Li, Y.; Xu, B.; Song, P.; Ma, F.; Sun, M., *J. Phys. Chem. C* **2017,** *121*, 12546-12561.

9. Song, P.; Li, Y.; Ma, F.; Pullerits, T.; Sun, M., *Chem. Rec.* **2016,** *16*, 734-753.

10. Voityuk, A. A., *J. Chem. Phys.* **2006,** *124*, 6.

11. Wang, Q. G.; Li, Y. Z.; Song, P.; Su, R. Z.; Ma, F. C.; Yang, Y. H., *Polymers* **2017,** *9*, 30.

12. Kjellberg, P.; He, Z.; Pullerits, T., *J. Phys. Chem. B* **2003,** *107*, 13737-13742.

13. Song, P.; Li, Y.; Ma, F.; Pullerits, T.; Sun, M., *J. Phys. Chem. C* **2013,** *117*, 15879-15889.

14. Wen, K. K.; Pan, X.; Feng, S. Y.; Wu, W. P.; Guo, X. G.; Zhang, J. L., *Mol. Phys.* **2019,** *117*, 303-310.

15. Bredas, J. L.; Beljonne, D.; Coropceanu, V.; Cornil, J., *Chem. Rev.* **2004,** *104*, 4971-5003.

16. Leng, C.; Qin, H.; Si, Y.; Zhao, Y., *J. Phys. Chem. C* **2014,** *118*, 1843-1855.

17. Zhang, X.; Chi, L.; Ji, S.; Wu, Y.; Song, P.; Han, K.; Guo, H.; James, T. D.; Zhao, J., *J. Am. Chem. Soc.* **2009,** *131*, 17452.

18. Li, Y.; Pullerits, T.; Zhao, M.; Sun, M., *J. Phys. Chem. C* **2011,** *115*, 21865-21873.

19. Bredas, J. L.; Calbert, J. P.; da Silva Filho, D. A.; Cornil, J., *Proc. Natl. Acad. Sci. USA* **2002,** *99*, 5804-5809.

20. Lan, Y. K.; Huang, C. I., *J. Phys. Chem. B* **2008,** *112*, 14857-14862.

21. Green, M. A., Solar cell fill factors: **1981,** *24*, 788-789.

22. Zhang, L.; Shen, W.; He, R.; Liu, X.; Tang, X.; Yang, Y.; Li, M., *Org. Electron.* **2016,** *32*, 134-144.

23. Liu, X.; Du, X.; Wang, J.; Duan, C.; Tang, X.; Heumueller, T.; Liu, G.; Li, Y.; Wang, Z.; Wang, J.; Liu, F.; Li, N.; Brabec, C. J.; Huang, F.; Cao, Y., *Adv. Energy Mater.* **2018,** *8*, 1801699.
